# FAMILY HEALTH EQUITY CONCEPTUAL MODEL

**DOI:** 10.1093/geroni/igae098.0700

**Published:** 2024-12-31

**Authors:** Genevieve Aguilar

**Affiliations:** University of Washington, Seattle, Washington, United States

## Abstract

Family Health Equity applies to the family unit when engaging in health care. The family (chosen and biological) unit is often the center of support for Black, Indigenous, and People of Color (BIPOC) communities. I am passionate about fully incorporating the patient and family into the care plan. This is especially true if there are barriers to care. Growing up along the US-Mexico border, I saw many barriers to care for my family. I propose this conceptual model of Family Health Equity. In this model, the patient has an equal voice and power to the caregiver and the provider. Other Health Equity models do not put the patient and caregiver equally at the center. This model starts with nursing driving the change and builds from there. The Family Health Equity conceptual model includes communication as a key element. This means including health literacy, interpretation, translation, identity, and cultural consideration in communication practices that can improve health equity for BIPOC communities. Any statements made by the patients or caregivers should be treated with respect and as truth. The outcome of these practices is an increased level of trust, which can lead to improved care. Studies suggest that patients have improved communication and experience fewer health disparities with racially concordant healthcare providers. The Family Health Equity conceptual model can be utilized to examine issues in family theory, aging, and clinical practice. It provides a blueprint for institutions to address equity in their environments.

